# Accuracy of CCL20 expression level as a liquid biopsy-based diagnostic biomarker for ovarian carcinoma

**DOI:** 10.3389/fonc.2022.1038835

**Published:** 2022-10-27

**Authors:** Watchara Sakares, Wannaporn Wongkhattiya, Ponlawat Vichayachaipat, Chompunoot Chaiwut, Varalee Yodsurang, Pattiya Nutthachote

**Affiliations:** ^1^ Department of Pharmacology and Physiology, Faculty of Pharmaceutical Sciences, Chulalongkorn University, Bangkok, Thailand; ^2^ Preclinical Toxicity and Efficacy, Assessment of Medicines and Chemicals Research Unit, Chulalongkorn University, Bangkok, Thailand; ^3^ Department of Obstetrics and Gynecology, Faculty of Medicine, Srinakharinwirot University, Bangkok, Thailand

**Keywords:** ovarian neoplasms, chemokine CCL2, chemokine CCL15, chemokine CCL20, chemokine CXCL14, biomarker

## Abstract

**Objective:**

The study aimed to investigate the potentiality of chemokines, including MCP-1, CCL15, CCL20, and CXCL14, as biomarkers for differential diagnosis between benign tumors and ovarian cancer (OC).

**Methods:**

A cross-sectional study was conducted in women aged >18 years who had adnexal masses treated with elective surgery at the HRH Maha Chakri Sirindhorn Medical Center, Srinakharinwirot University, between 2020 and 2021. The preoperative MCP-1, CCL15, CCL20, and CXCL14 serum levels were measured using a sandwich enzyme-linked immunosorbent assay. Preoperative diagnosis was defined according to the risk of malignancy index. The histological diagnosis and cancer subtype were confirmed using pathological specimens.

**Results:**

Ninety-eight participants were preoperatively diagnosed with malignant tumors. The pathological diagnosis confirmed OC in 33 patients and disclosed 27 misdiagnosed cases, of which endometriotic cyst was the most common (44.44%). CCL20 and CA125 serum levels were significantly higher in the patients with cancer than in those with benign. In addition, CCL20 level could differentiate between benign and early-stage malignancy. Furthermore, only CCL20 levels could distinguish endometriotic cysts from OC, whereas CA125 levels could not. Concordant with the serum protein level, the increased mRNA level of *CCL20* was observed in ovarian cancers comparing with that in benign tissues. We found that CCL20 levels could differentiate between benign tumors and OC with 60.61% sensitivity and 75.44% specificity at the optimal cutoff value of 38.79 pg/ml. Finally, the logistic regression model integrating CCL20, CA125, and menopause status promoted diagnostic accuracy by increasing the specificity to 91.23%.

**Conclusions:**

Our study revealed the potential usefulness of CCL20 level as a biomarker for diagnosing early-stage OC with endometriosis differentiation. We recommend further studies to confirm the accuracy of CCL20 levels with the current diagnosis in a large patient sample.

## Introduction

Ovarian cancer (OC) is the second most common gynecological cancer, with a reported incidence rate of 7.9 per 100,000 women annually (1). It is the leading cause of cancer death among women in developed countries, including Thailand (2, 3). Its fatality and 5-year survival rates are approximately 70% and 28%, respectively (4). Almost 75% of OC cases are diagnosed in the advanced disease stage (III/IV), when transperitoneal, hematogenous, and/or lymphatic spread have already occurred, leading to poor prognosis and high recurrence rates (5). Epithelial OC (EOC) is the most common type that can be classified histologically as high- and low-grade serous, mucinous, clear cell, and endometrioid carcinomas (1). The standard treatment for OC is surgical removal of the tumor, followed by first-line platinum-based and paclitaxel chemotherapy (6). Although serum carbohydrate antigen 125 (CA125) and ultrasonography are the most widely used diagnostic tools for OC, they demonstrate low sensitivity and specificity for the early detection of OC. Elevated CA125 levels are not specific to OC and also occur in non-cancer patients with endometriosis inflammation and patients with other cancer types (7, 8). In addition, approximately 20% of patients with OC have normal CA125 levels (9). Therefore, the current research focuses on novel diagnostic biomarkers that show high accuracy and specificity for OC (10, 11).

Inflammatory conditions increase the risk of developing OC (12). Inflammation can activate transcription factors such as nuclear factor-κB, signal transducer and activator of transcription 3, and hypoxia-inducible factor 1α, which are associated with inflammatory mediators, prostaglandins, cytokines, and chemokine production in tumor cells. These processes activate various inflammatory cells and change the tumor microenvironment, promoting malignant generation (13). Chemokines are small cytokines secreted during the inflammatory process to regulate immune cells. Chemokines, which consist of four conserved cysteine residual domains linked by a disulfide bond, are divided into four groups according to the position of these cysteines: C, CC, CXC, and CX3C (14, 15). Chemokines can induce the migration of leukocytes, including monocytes, lymphocytes, granulocytes, natural killer cells, and tumor-associated macrophages (TAMs), into pathological conditions, including infections, inflammatory reactions, and tumors. Chemokines affect tumor progression *via* several mechanisms, including angiogenesis, cell proliferation, migration, and immune invasion, which are necessary to ensure the success of growing tumors and disseminating metastasis (15). Angiogenesis is an important condition in the progression of cancers, including ovarian carcinoma (16). Several CXC chemokines have been reported to induce either endothelial cell migration or proliferation and neovascularization and to play roles in tumor growth. CC chemokines have been reported to play both direct and indirect roles in angiogenesis (17). The angiogenic response induced by CCL2 was accompanied by an inflammatory response and induced chemotaxis of human endothelial cells and the formation of blood vessels (18).

Several chemokines have been explored for their potential usefulness in the diagnosis and prognosis of OC (19, 20). Previous studies have shown that elevated levels of some chemokines, including CCL2 or MCP-1 (21), CCL15, CCL20 (22), and CXCL14 (23), were detected in serum samples from patients with OC. However, these potential biomarkers have never been validated as preoperative diagnostic markers. The aim of this study was to evaluate the diagnostic accuracy and optimal cutoff values of CCL2, CCL15, CCL20, and CXCL14 compared with CA125 for OC diagnosis.

## Materials and methods

### Ethics statement

This cross-sectional study was conducted at the Department of Obstetrics and Gynecology, HRH Maha Chakri Sirindhorn Medical Center, Srinakharinwirot University, with the approval of the institutional review board (certificate No. SWUEC/F-047/2563). Written informed consent was obtained from all participants. All procedures were performed in accordance with the relevant guidelines and regulations. Research staff members had completed the Good Clinical Practice training.

### Patients

The inclusion criteria were women with clinically diagnosed ovarian or adnexal masses who underwent elective surgery between July 2020 and October 2021, with ages >18 years, and with an American Society of Anesthesiologists physical status score of I–II. Participants were excluded if they had a history of chemotherapy or radiotherapy, serious psychiatric disease, pregnancy, previous history of OC or any malignancy, active inflammatory disorders, i.e., rheumatoid arthritis, multiple sclerosis, and type I diabetes, and refused to sign an informed consent form. Patients without intraoperative findings indicating a pelvic or adnexal mass were also excluded from the data analysis. All participants were hospitalized for preoperative preparation at least 24 hours before surgery, and a blood sample was preoperatively collected *via* a peripheral venous puncture. Clinical data, preoperative diagnosis, operative procedure, and postoperative diagnosis were recorded. Preoperative diagnosis was defined according to the risk of malignancy index (RMI) based on the score calculated from the serum CA125 level, menopause status, and ultrasonography. Postoperative diagnosis was based on pathologists’ histological interpretations.

### Quantification of biomarkers

The clot blood sample was centrifuged at 3,000 RPM for 10 minutes to separate serum immediately and stored at −20°C until the analysis. Chemokines were quantitatively analyzed in patient serum using a sandwich enzyme-linked immunosorbent assay (ELISA) kit from R&D Systems (catalog Nos. DY279, DY360, DY363, and DY866 for MCP-1, CCL20, CCL15, and CXCL14, respectively), in accordance with the manufacturer’s instructions. The absorbance was determined instantly using a CLARIOstar Plus microplate reader (BMG LABTECH, Germany) at wavelengths of 450 and 570 nm. The concentration of each chemokine was calculated by subtracting each absorbance reading at 570 nm from the reading at 450 nm and the average of duplicate readings, and then normalized with the average of the blanks. A standard curve was created with a quadratic polynomial fitting curve in Microsoft Excel for MAC version 16.58. The standard curve was considered as best fitted if *r* ^2^ value of the fitting line was >0.98. The CA125 level was obtained from routine hospital laboratory measurements.

### Differential expression analysis

The mRNA expression levels of *CCL20* and *CA125* (*MUC16*) in human ovarian tissues were retrieved from two public datasets, GSE4122 (24) and GSE17308 (25), available in NCBI’s Gene Expression Omnibus, which included 46 and 67 patients, respectively. We categorized the patient tissues, based on the pathological diagnosis, into 3 groups, i.e., benign, borderline ovarian tumors (BOTs), and malignancy groups.

### Statistical analysis

Statistical analysis was performed using a power of 80% and an *α* value of 0.05. Chemokines with concentrations below the detection limit were excluded from the data analysis. The patients’ baseline characteristics were presented as frequency and percentage or mean and standard deviation. Categorical data, including patient and operation characteristics, were analyzed using the chi-square test. Receiver-operating characteristic (ROC) curves were plotted, and the area under the curve (AUC) was calculated to compare the performance of the biomarkers for predicting OC in terms of sensitivity, specificity, positive predictive value, and negative predictive value. The ROC curves were compared using a chi-square test. The web tools easyROC version 1.3.1 and STATA 14.1 (StataCorp. 2015. Stata Statistical Software: Release 14. College Station, TX: StataCorp LP) were used to calculate the optimal cutoff values (Youden index) (26). A logistic regression analysis was performed for the preoperative factors. The preoperative factors were integrated into the model if the model’s parameter p-value was <0.05 and excluded from the model if the p-value was >0.1. P-values <0.05 were considered statistically significant. A statistical analysis was performed using GraphPad Prism version 9.3.1 (350) for macOS Monterey (GraphPad Software, San Diego, California USA, https://www.graphpad.com) and Statistical Package for Social Sciences (SPSS) versions 28 for Windows (SPSS, Chicago, IL, USA).

## Results

### Patients’ characteristics and diagnoses

This study included 100 patients for eligibility. Two patients with gastrointestinal stromal tumors were excluded from the analysis; thus, 98 patients were included in the analysis, as shown in **Table 1**. We used the RMI to determine the preoperative risk of malignancy. Forty patients (40.82%) were preoperatively diagnosed with benign disease; and 58 patients (59.18%), with malignancy. However, the pathological diagnosis indicated that 59 patients (60.20%) had benign tumors, 6 (6.13%) had BOTs, and 33 (33.67%) had OCs. We found a discrepancy between the preoperative and postoperative diagnoses in 27 patients (27.55%), and 23 histologically confirmed benign cases were preoperatively diagnosed as malignant. Endometriotic cyst was the most common misdiagnosis, with up to 12 misdiagnosed patients (44.44%).

**Table 1:** Baseline characteristics and histopathology of the participants who presented with pelvic and adnexal masses.

Variable	Total (N = 98)
Clinical characteristics	
Age (years), mean (SD)	47.06 (15.55)
Parity, n (%)	
0	51 (52.04)
1	16 (16.33)
2	21 (21.43)
≥3	10 (10.20)
Menopausal status, n (%)	
Premenopausal	67 (68.37)
Postmenopausal	31 (31.63)
Histopathology, n (%)	
1. Benign gynecological disease/benign tumors	59 (60.20)
−Functional/simple cyst	6 (10.17)
−Serous cystadenoma	12 (20.34)
−Mucinous cystadenoma	4 (6.78)
−Teratoma	4 (6.78)
−Endometriotic cyst	31 (52.54)
−Tubo-ovarian abscess	2 (3.39)
2. Borderline ovarian tumors, n (%)	6 (6.12)
3. Cancer subtype, n (%)	33 (33.67)
3.1. Epithelial ovarian cancer	23 (69.70)
−Serous carcinoma	13 (56.52)
−Mucinous cystadenoma	4 (17.39)
−Endometrioid carcinoma	2 (8.70)
−Clear cell carcinoma	4 (17.39)
3.2 Nonepithelial ovarian cancer	10 (30.30)
Tumor stage	
Stage I	15 (45.45)
Stage II	5 (15.15)
Stage III	12 (36.36)
Stage IV	1 (3.03)
Operation, n (%)	
Unilateral SO^*^/cystectomy	32 (32.65)
TAH with BSO^†^	12 (12.24)
TAH with BSO^†^ with complete surgical staging	54 (55.10)

^*^Salpingo-oophorectomy.

^†^Total hysterectomy with bilateral salpingo-oophorectomy.

### Chemokine levels in benign and malignant cases

**Figure 1:**
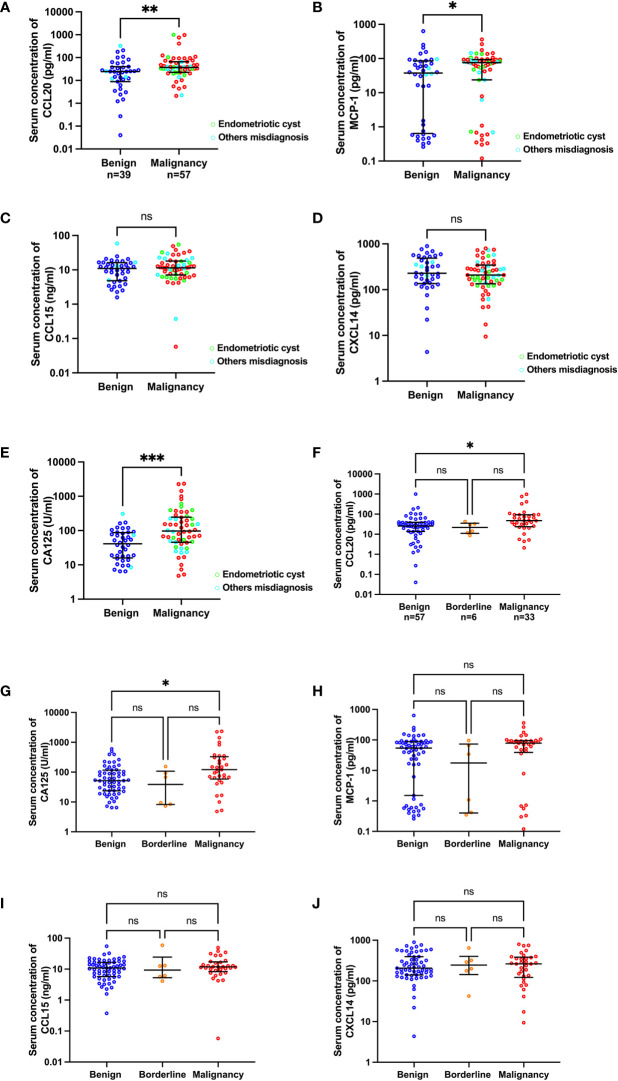
Scatter plots of CA125 and chemokine concentrations categorized by preoperative diagnosis **(A–E)** and pathological diagnosis **(F–J)**. The green and cyan circles in **(A–E)** indicate preoperatively misdiagnosed cases of endometriotic cyst and others, respectively.*p<0.05; **p<0.01; ***p<0.001; not significant (ns). The lower and upper whiskers are interquartile ranges, and the middle line is the median.

To determine the preoperative protein levels in the patients’ serum samples, an ELISA kit was used to measure the concentrations of MCP-1, CCL15, CCL20, and CXCL14 chemokines. A total of 98 samples were analyzed for MCP-1, CCL15, and CXCL14. Two samples had CCL20 concentrations below the detection limit, leaving 96 samples analyzed for this chemokine. The concentrations of MCP-1 and CCL20 in the preoperatively diagnosed malignant group were significantly higher than those in the benign group (median [IQR], pg/ml: MCP-1, 75.46 [23.772–92.71] vs 37.67 [0.65–85.58], p=0.041 and CCL20, 36.83 [22.58–65.76] vs 24.35 [8.84–40.19], p=0.009; **Figures 1A, B**). CCL15 and CXCL14 levels were not statistically different between the malignant and benign groups (**Figures 1C, D**). As expected, the CA125 level (U/ml) significantly increased in the malignant group compared with the benign group (97.70 [5.65–245.80] vs 41.30 [15.88–87.43], p=0.0002; **Figure 1E**). Next, we analyzed the chemokine concentrations in the samples categorized according to histologically confirmed diagnosis to identify the potential biomarker that could predict pathological results before surgery (**Table 2**). The results showed that among the 4 chemokines, only CCL20 showed significantly different levels (pg/ml) between the benign and malignant groups (25.64 [13.59–39.32] vs 47.47 [23.69–93.30], p=0.015; **Figure 1F**). As a control, the CA125 level (U/ml) was significantly different between the benign and malignant groups (51.70 [23.70–117.00] vs 122.00 [58.80–329.50], p=0.021; **Figure 1G**). However, the chemokines and CA125 could not differentiate the BOTs in this study (**Table 2** and **Figures 1F–J**).

**Table 2:** Serum concentrations of potential biomarkers in the different pathological subgroups.

Potential biomarker (unit)	Median serum concentration (interquartile range) (N = 98)		
	Benign (n = 59)	Borderline (n = 6)	Malignancy (n = 33)
CA125 (U/ml)	51.70 (23.70–117.00)	38.66 (8.21–107.50)	122.00 (58.80–329.50)*
CCL20^†^ (pg/ml)	25.64 (13.59–39.32)	21.75 (11.06–35.35)	47.47 (23.69–93.30)^*^
MCP-1 (pg/ml)	53.86 (1.51–88.04)	17.59 (0.40–79.48)	78.34 (38.92–94.70)
CCL15 (ng/ml)	10.81 (5.74–16.59)	9.33 (5.25–24.37)	11.79 (8.30–17.07)
CXCL4 (pg/ml)	206.01 (140.21–394.75)	244.73 (143.57–401.80)	262.66 (121.87–384.03)

^*^Comparison with the benign group, p< 0.05

^†^Total for CCL20, n = 9 6: benign, n = 57; borderline, n = 6; and malignancy, n = 33.

### CCL20 levels in endometriosis differentiation

**Figure 2:**
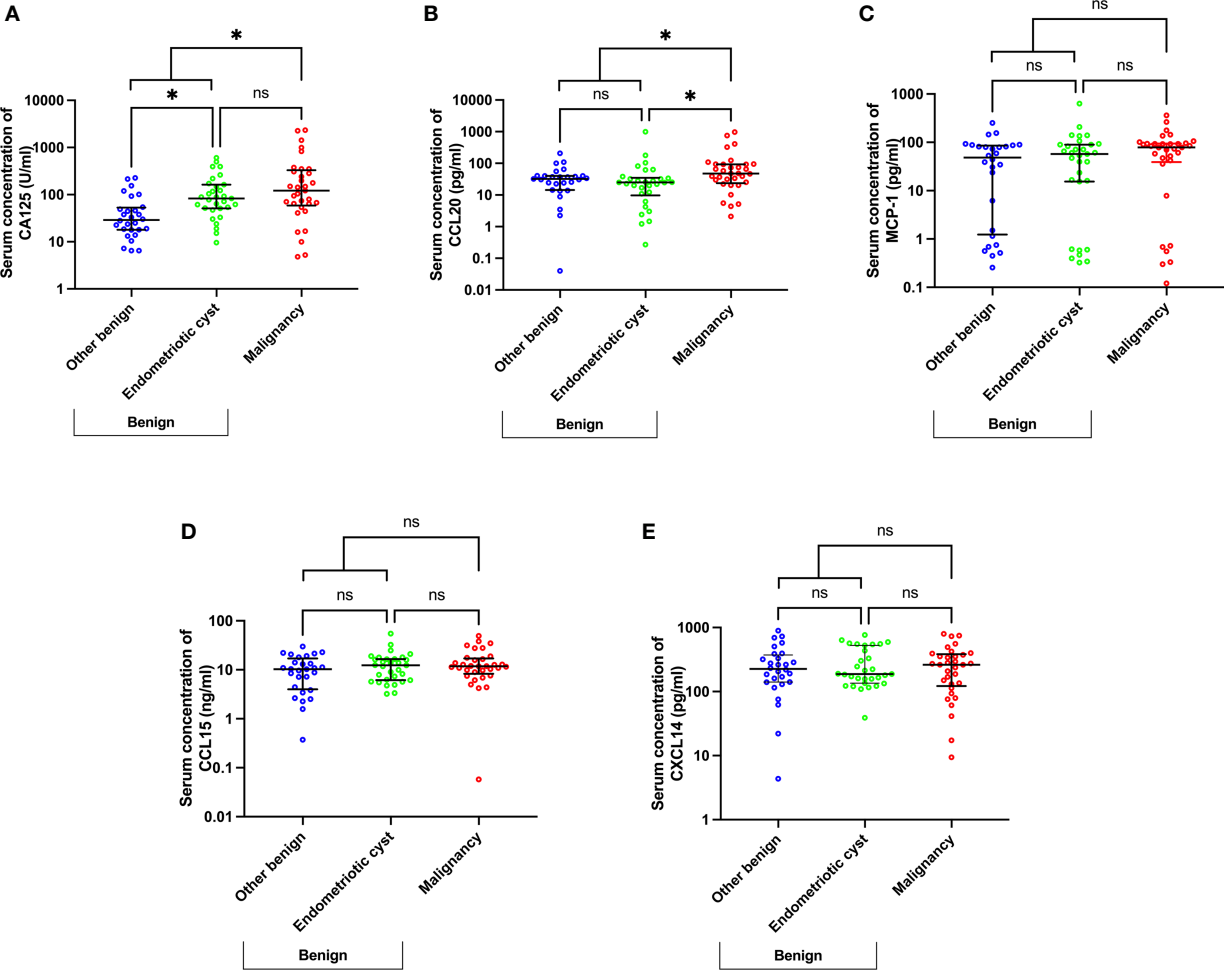
Scatter plots of CA125 **(A)** and chemokine **(B–E)** concentrations using a subgroup analysis of benign cases, namely endometriotic cysts, other benign diseases, and malignancy. *p<0.05; not significant (ns). The lower and upper whiskers are interquartile ranges, and the middle line is the median.

To disclose the potential biomarkers that can distinguish between patients with malignancy and endometriotic cysts, chemokine levels were further analyzed in malignant cases compared with endometriotic and other benign cases, which were classified according to pathological results (**Figure 2**). By comparing with other benign cases (median [IQR], U/ml: 29.00 [17.93–53.15]), CA125 levels (U/ml) were found to be significantly elevated in the endometriotic cysts and malignant cases (82.70 [51.00–163.00], p=0.015 and 122.00 [58.80–329.50], p=0.001, respectively) and were not statistically different between the endometriotic cysts and malignant cases (**Figure 2A**). Of interest, the CCL20 level (pg/ml) in endometriosis (24.86 [9.74–35.00]) was comparable with that in other benign cases (32.12 [14.33-40.20]) and significantly decreased compared with that in malignant cases (47.47 [23.69–93.30]), with a p-value of 0.019 (**Figure 2B**). In consideration of the misdiagnosed subgroup, the endometriotic cysts exhibited remarkably increased CA125 levels (U/ml: 183.50 [48.90–394.30]) compared with the other benign cases (46.00 [19.00–92.00], p=0.002), but the CCL20 levels (pg/ml) were not significantly different (32.28 [22.05–38.45] vs 34.50 [1.15–65.44]), respectively; **Figure S1**). These results suggest that high CA125 levels in endometriotic cases can lead to misdiagnosis, and measurement of CCL20 levels can potentially solve this problem.

### CCL20 level in tumor stage differentiation

**Figure 3:**
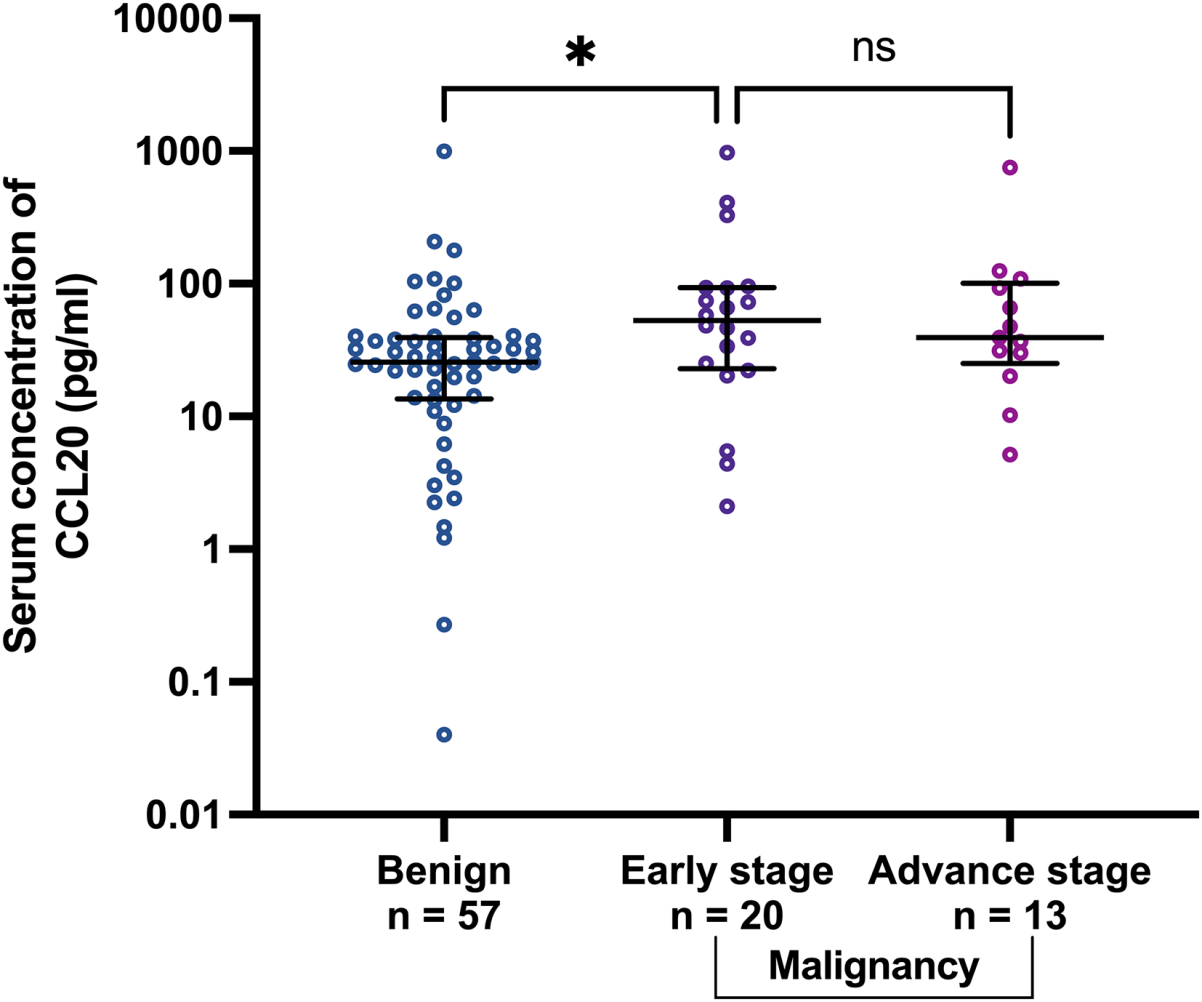
Scatter plots of CCL20 concentrations in benign group comparing with early stage (stage I and II) and advanced stage (stage III and IV) malignancies. *p<0.05; not significant (ns). The lower and upper whiskers are the interquartile ranges, and the middle line is the median.

To investigate whether CCL20 could differentiate between benign and early-stage malignancy, the CCL20 levels were subgroup analyzed by tumor stage, i.e., early stage (stages I and II) and advanced stage (stages III and IV). CCL20 levels (pg/ml) significantly increased in early-stage tumor than in benign cases (median [IQR], 53.02 [22.97–93.43] and 25.64 [13.59–39.32], p=0.045, respectively) (**Figure 3**). CA125 level demonstrated no statistically significant difference between benign cases and early-stage OCs (median [IQR], 51.70 [23.70–117.00], and 132.4 [4.79–302.40], respectively) (**Figure S2**).

### *CCL20* differential gene expression

**Figure 4:**
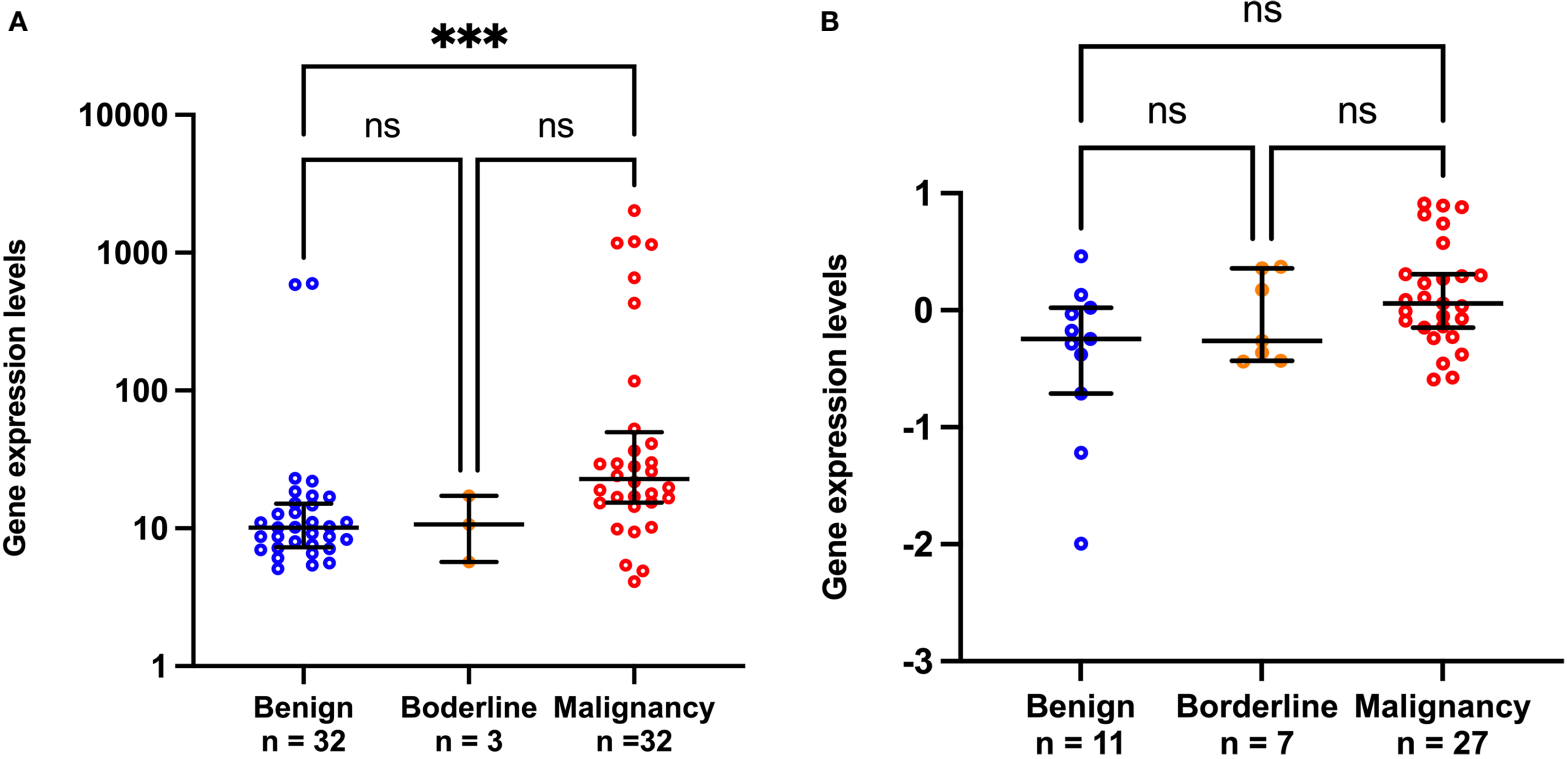
Scatter plots of *CCL20* gene expression from public GSE4122 dataset **(A)** and GSE17308 dataset **(B)**. ***p<0.001; not significant (ns). The lower and upper whiskers are interquartile ranges, and the middle line is the median.

We further validated our finding using two public patient datasets which had available data of the mRNA expression level of *CCL20* and pathological diagnosis results. GSE4122 dataset showed that *CCL20* level significantly increased in the malignancy compared to benign groups (p<0.001; **Figure 4A**), in contrast to *CA125* level which significantly decreased in the malignancy compared to the benign groups (p<0.0001; **Figure S3A**). GSE17308 dataset showed a trend of high *CCL20* level in malignancy, though the difference between benign and malignancy did not achieve the significance level (**Figure 4B**), whereas *CA125* level increased in malignancy compared to benign groups (**Figure S3B**).

### Diagnostic accuracy of CCL20 levels

**Figure 5:**
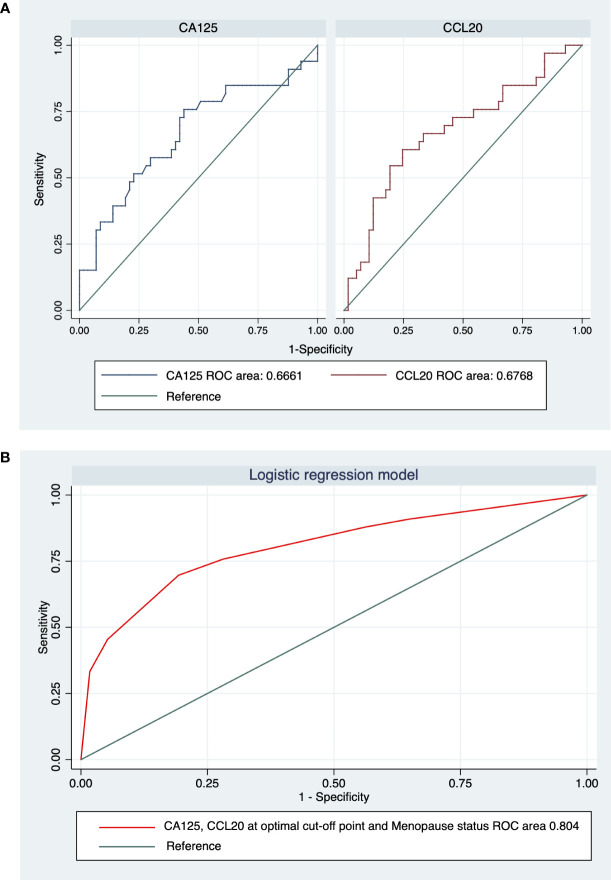
Receiver-operating characteristic (ROC) curve analysis and areas under the curve (AUC) for **(A)** CA125 and CCL20 and **(B)** the logistic regression model integrating CA125, CCL20, and menopause status.

A ROC analysis was performed to demonstrate the pathological diagnostic performances of CA125 and CCL20 levels as potential biomarkers of OC (**Figure 5**). The areas under the ROC curve of CA125 and CCL20 levels for differentiating malignant from benign (n=90; excluded 6 BOTs and 2 samples below the detection limit) were 0.666 (95% confidence interval [CI], 0.544–0.788) and 0.677 (0.558–0.796), respectively (**Figure 5A**). The areas under the 2 ROC curves were not significantly different (p=0.895). The optimal cutoff value defined using the Youden index method, to distinguish between benign and malignant tumors was 62.65 U/ml for CA125 and 38.79 pg/ml for CCL20. The sensitivity, specificity, and positive and negative predictive values at these cutoff points of the ROC curves are shown in **Table 3**. Concisely, CCL20 exhibited higher specificity (75.44% vs 56.14%), improved diagnostic accuracy (70.00% vs 63.33%), and lower sensitivity (60.61% vs 75.76%) compared with CA125 at the optimal cutoff point. Logistic regression was used to build a diagnostic model to identify preoperative factors that could improve diagnostic accuracy. The model proposed three predictors, namely postmenopausal status, CA125 level, and CCL20 level, with odds ratios (95% CI) of 5.85 (1.99–17.18), 3.63 (1.22–10.79), and 3.12 (1.11–8.77), respectively. The model demonstrated 51.52% sensitivity and 91.23% specificity, with 76.76% diagnostic accuracy **Table 3** and **Figure 5B**).

**Table 3:** Diagnostic performances and optimal cutoff values of serum CA125 and CCL20 levels for discriminating between benign disease and ovarian cancer.

Performance measure	CA125	CCL20	Logistic regression model CA125 + CCL20 + postmenopause
Youden optimal cutoff value	62.65 U/ml	38.79 pg/ml	–
Sensitivity (%)	75.76 (57.70–88.90)	60.61 (52.10–77.10)	51.52 (33.50–69.20)
Specificity (%)	56.14 (42.40–69.30)	75.44 (62.20–85.90)	91.23 (80.70–97.10)
Positive predictive value (%)	50.00 (35.50–64.50)	58.82 (40.70–75.40)	77.27 (54.60–92.20)
Negative predictive value (%)	80.00 (64.40–90.90)	76.79 (63.60–87.00)	76.47 (64.60–85.90)
Test accuracy (%)	63.33 (52.51–73.25)	70.00 (59.43–79.21)	76.67 (66.57–84.94)

The 95% confidence interval (CI) is in parentheses.

## Discussion

The standard diagnosis of OC is based on serum CA125 levels, ultrasonography, and menopause status. Three of the 4 patients were diagnosed in the advanced stage and had a poor prognosis and increased mortality rate compared with early-stage patients (4–6). The CA125 level is the most widely used biomarker for diagnosis, monitoring treatment efficacy, and predicting the prognosis of OC (27). However, the CA125 level is not reliable for screening or early detection because of the high rates of false-positive and false-negative results and the many factors that affect CA125 levels, such as age, race, ethnicity, smoking history, and obesity (28). Elevated CA125 levels could be found in patients with benign conditions (29); this raises the malignancy risk (RMI score) and leads to misdiagnosis. Of the 27 misdiagnosed patients in this study, 12 who had endometriosis (44.44%) were preoperatively diagnosed with malignancy and exhibited significantly high serum CA125 levels compared with those with other benign cases (**Figure S1A**). The percentage of misdiagnosed endometriosis in our study was comparable with that previously reported by Yamamoto et al. (30), who found endometriosis in 40% of false-positive cases using the RMI method. This non-specificity drawback of CA125 warrants the need for novel OC-specific diagnostic biomarkers.

CCL20 is a proinflammatory chemokine originating from T-helper 17 cells that is responsible for normal function of lymphocyte cells. CCR6 is a CCL20 receptor expressed on dendritic cells, T cells, and B cells (31). In cancer research, CCL20 plays a crucial role in neoplastic processes, and TAM is its major source. CCL20 and CCR6 in tumors can promote cell proliferation, migration, invasion, metastasis, and angiogenesis by directly stimulating vascular endothelial cells, thereby increasing vascular endothelial growth factor expression (32–34). CCL20 levels are elevated in many cancer types such as breast, liver, and pancreatic cancers, but are low in adrenal gland and lung cancers (31). In OC, it has been reported to contribute to promoting chemotherapy resistance, stimulating migration, and poor disease prognosis (35–37). Increased CCL20 levels were found in serum samples from patients with recurrent OC who were completely treated with platinum-based chemotherapy and correlated with decreased recurrence-free survival rates (19).

Our results showed that CCL20 levels could distinguish OC from benign diseases, including endometriotic cysts. The misdiagnosed patients with endometriosis demonstrated low serum CCL20 levels, similar to those in other benign cases, but showed remarkably high CA125 levels (**Figure S1B**). This results in improved specificity (75.44%) and diagnostic accuracy (70.00%) of CCL20 compared with those of CA125 (**Table 3**). Serum CA125 levels are elevated in women with cystic ovarian endometriosis and imitate those in OC, especially in premenopausal women (38, 39). By contrast, the serum CCL20 levels in premenopausal women have been reported to be lower in those with endometriosis than in those without endometriosis (40). Our results indicate the potential of usefulness of CCL20 level as a biomarker to differentiate endometriosis in the setting of ovarian tumors with elevated CA125 levels, which can reduce the false positivity rate from 41.11% in preoperative diagnosis to 15.56% at the optimal cutoff value of CCL20. This suggests that the factors used for preoperative diagnosis should be modified to improve diagnostic accuracy. Moreover, the CCL20 levels significantly increased in early-stage malignancy compared to benign. Whereas CA125 has low specificity to detect the early-stage OC in our study and in previous publications (41–43). These suggest the advantage of using CCL20 level as a biomarker for early OC detection. We further validated the plasma protein level of CCL20 using differential gene expression analysis in benign and malignant tissues. Although there was a limited number of patient tissues, the *CCL20* gene expression was significantly high in malignant tissues comparing with that in benign tissues. These consistent expressions support the theory that CCL20 is a proinflammatory cytokine which is excreted by tumor cells and involved with early tumor progression (44).

The accuracy of the diagnostic biomarker was calculated using ROC analysis, and the results suggested that the optimal cutoff value of CA125 was 62.65 U/ml, with 75.76% sensitivity and 56.14% specificity (**Table 3**). Maggino et al. (45) reported that the cutoff value of CA125 was 65 U/ml, with 71.7% sensitivity and 92.5% specificity. CA125 showed lower sensitivity (60%) and specificity rates (89%) at a cutoff value of 65 U/ml in a subgroup of premenopausal patients (46) compared with postmenopausal women (78% sensitivity and 97% specificity). A possible explanation for the low specificity of CA125 in our study is that almost 70% of our population were premenopausal and up to 60% had benign disease. The ROC analysis revealed that using either CA125 or CCL20 could not differentiate OC in all cases. Thus, we generated a logistic regression model that finally integrated three significant predictors of OC, namely postmenopausal status and CA125 and CCL20 levels at optimal cutoff values. The model combining these three parameters exhibited high specificity (91.23%), with a sensitivity of 51.52% and an accuracy of 76.76%. Postmenopausal status had the highest probability (5.85 times) of predicting OC, followed by CA125 and CCL20 levels (3.63 and 3.12 times, respectively). The final model increased the probability of diagnosing OC to up to 11 times, indicating that the combination of CCL20 measurements and the diagnostic index led to a more efficient diagnosis with improved specificity to OC than the standard RMI. These results indicate the diagnostic potential of CCL20 as a specific biomarker and preoperative diagnostic tool for OC. To our knowledge, CCL20 has never been studied for its role as a diagnostic biomarker for OC.

Owing to the limited number of patients with OC in this study (n=33), we did not find any candidate biomarkers for the histological subtypes of OC (data not shown). The only tissue subtype that could be differentiated was the tissue origin, that is, epithelial, or nonepithelial cells, based on the elevated CA125 levels in the epithelial subtype (**Figure S4**). This indicates the variability of CA125 levels among OCs, which could be further studied. Although only CCL20 showed potential as an upregulated biomarker for OC, all other chemokines tested in this study demonstrated trends of high serum levels in malignancy. We recommend performing further research on CCL20 with a larger sample size to confirm our findings and explore the new aspect of this cytokine, especially in women with endometriotic cysts. To investigate the additional role of CCL20 as a biomarker for predicting prognosis and treatment efficacy, we suggest further study to monitor the postoperative levels of chemokines, disease progression of patients, and treatment efficacy. Lastly, BOTs are still a problem in differential diagnosis; the chemokines or CA125 could not differentiate them from benign or malignant diseases, which suggests the need for an efficient diagnostic method to overcome this difficulty.

In conclusion, CCL20 and CA125 could be utilized as diagnostic biomarkers for OC, although CCL20 provides higher specificity to endometriotic disease, and early stage of OCs detection. However, the expression levels of CXCL14, CCL15, and MCP-1 are not suitable for predicting endometriosis, as they showed no significant difference between benign and malignant ovarian tumors. This finding must be validated in a larger number of patients with histologically confirmed OC. Testing for a novel biomarker in patients before surgery will be beneficial for choosing the most appropriate therapeutic options.

## Data availability statement

The datasets presented in this study can be found in online repositories. The names of the repository/repositories and accession number(s) can be found in the article/**Supplementary Material**.

## Ethics statement

The studies involving human participants were reviewed and approved by Institutional Review Board (IRB), Strategic Wisdom and Research Institute, Srinakharinwirot University. The patients/participants provided their written informed consent to participate in this study.

## Author contributions

WS, WW, PV, and CC performed formal analysis and investigation. WS also performed data curation, statistical analysis, and visualization. WS wrote the first draft of the manuscript. VY supervised formal analysis and investigation. PN contributed the resources. VY and PN contributed to the conceptualization, methodology, design of the study, validation of the results, funding acquisition, and reviewing and editing of the manuscript. All authors contributed to the article and approved the submitted version.

## Funding

This study was granted by Research Fund, the Faculty of Medicine, Srinakharinwirot University and supported by The Second Century Fund (C2F), Chulalongkorn University

## Acknowledgments

All members of Department of Obstetrics and Gynecology, Faculty of Medicine, Srinakharinwirot University. WS and VY are supported by The Second Century Fund (C2F), Chulalongkorn University.

## Conflict of interest

The authors declare that the research was conducted in the absence of any commercial or financial relationships that could be construed as a potential conflict of interest.

## Publisher’s note

All claims expressed in this article are solely those of the authors and do not necessarily represent those of their affiliated organizations, or those of the publisher, the editors and the reviewers. Any product that may be evaluated in this article, or claim that may be made by its manufacturer, is not guaranteed or endorsed by the publisher.
