# Supplementary material for: Accuracy of CCL20 expression level as a liquid biopsy-based diagnostic biomarker for ovarian carcinoma
Source: Front Oncol. 2022 Oct 27;12:1038835. doi: 10.3389/fonc.2022.1038835 (PMC9647055; doi:10.3389/fonc.2022.1038835)
Supplement: Supplementary file 1 [file DataSheet_1.pdf]

Supplementary material

Supplementary Figure S1

Evaluation of CA125 levels leads to misdiagnosis because non-cancer patients with endometriosis also have high CA125 levels. Thus, the measurement of CA125 levels could lead to false-positive ovarian cancer (OC), which decreases the specificity of the biomarker. CCL20 levels were not significantly different between all four groups, which suggests the constancy of CCL20 levels in all benign cases and the potential of CCL20 as a specific biomarker of OC.

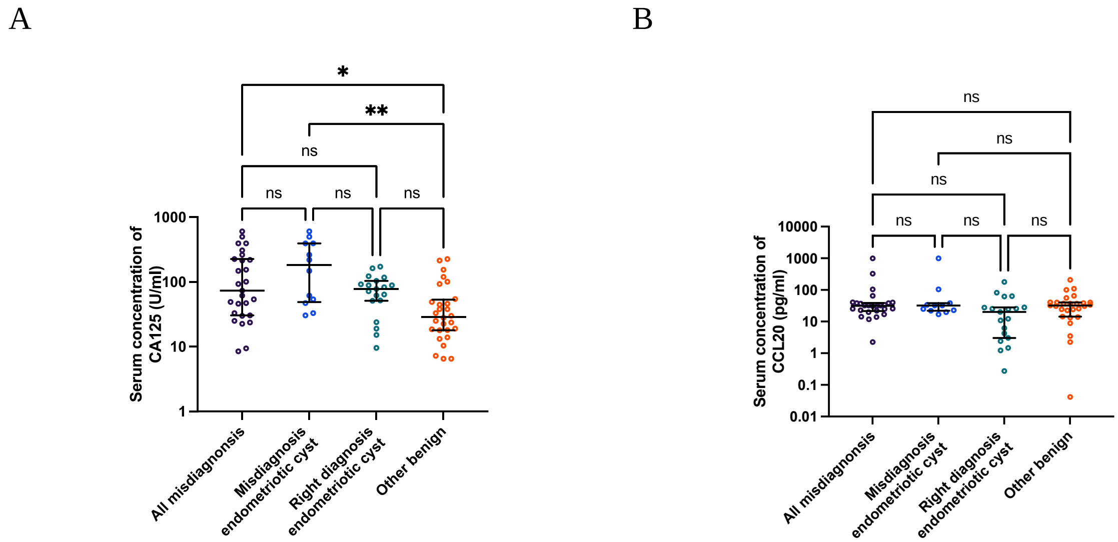

Figure S1. Scatter plot of CA125 and CCL20 serum concentrations in all cases of misdiagnosis, misdiagnosed endometriosis, right diagnosis of endometriosis, and other benign diseases. *p <0.05; **p <0.01; not significant (ns). The lower and upper whiskers are the interquartile ranges, and the middle line is the median

Supplementary Figure S2

The comparison between benign and malignancy groups which is a subgroup to early stage malignancy (stage I and II) and advanced stage malignancy (stage III and IV) show CA125 level show differences among three groups (p=0.019) but cannot identify which group was a significant difference (51.70 [23.70–117.00], 132.4[4.79–302.40], and 104.00 [55.96–603.0] for benign, early stage, and advanced stage malignancy, respectively;)

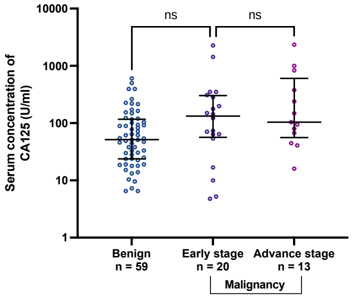

Figure S2. Scatter plots of CA125 concentrations in benign comparing with early and advanced malignancy stage. The lower and upper whiskers are the interquartile ranges, and the middle line is the median, not significant (ns).

Supplementary Figure S3

The comparison of *CA125* (*MUC16*) gene expression using the retrieved mRNA expression data from two public datasets, GSE17308 and GSE4122, of NCBI's Gene Expression Omnibus database. GSE4122 dataset, the *CA125* expression level reduced significantly from the benign to malignancy group and in GSE17308 dataset the expression level was significantly increased in malignancy and borderline groups compared to the benign group.

A GSE4122 **B** GSE17308

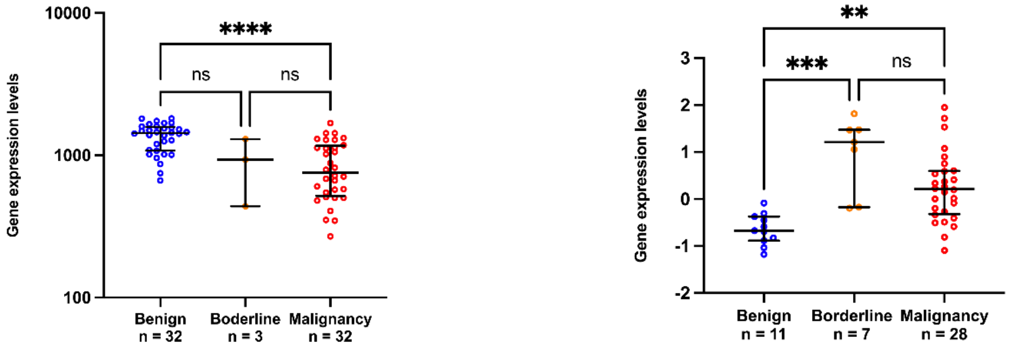

Figure S3. Scatter plots of *CA125* gene expression from public GSE4122 dataset (left panel) and GSE17308 dataset (right panel). **p <0.01; ***p <0.001; ****p <0.0001; not significant (ns). The lower and upper whiskers are interquartile ranges, and the middle line is the median.

Supplementary Figure S4

The comparison between the epithelial and nonepithelial malignancy cell subtypes showed that only the CA125 levels were significantly increased in the epithelial cells (median [interquartile range, IQR], 150 U/ml [79.60–376.00 U/ml]) compared with the nonepithelial cells (58.80 U/ml [34.69–138.00 U/ml], $p = 0.02$). The levels of the other chemokines were not significantly different between the epithelial and nonepithelial subtypes, as follows: CCL20 (47.47 pg/ml [20.27–93.82 pg/ml] vs 47.38 pg/ml [28.09–81.89 pg/ml]), MCP-1 (78.34 pg/ml [42.08–106.10 pg/ml] vs 80.70 pg/ml [6.08–94.03 pg/ml]), CCL15 (11.97 ng/ml [7.50–16.54 ng/ml] vs 11.41 ng/ml [9.31–21.98 ng/ml]), and CXCL14 (262.70 pg/ml [149.70–423.7 pg/ml] vs 203.60 pg/ml [78.30–309.30 pg/ml]).

A

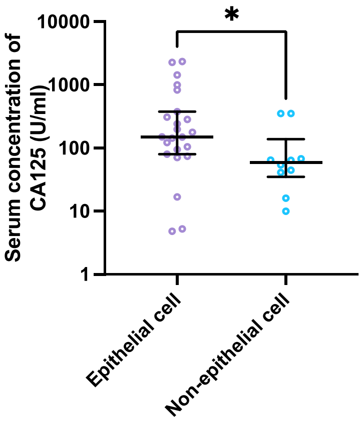

B

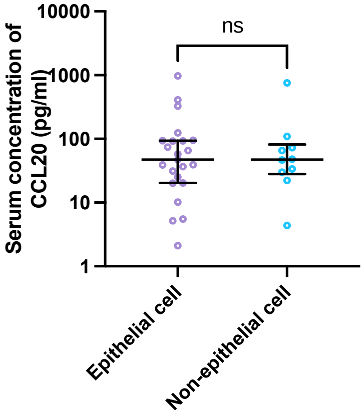

C

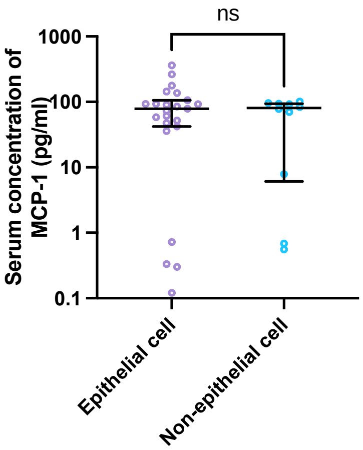

D

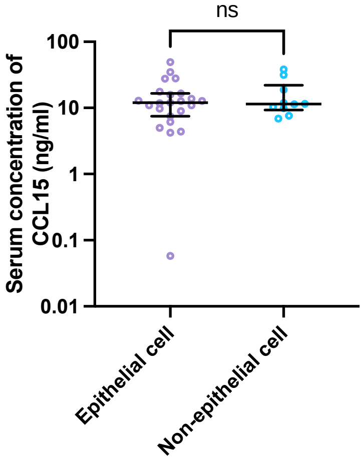

E

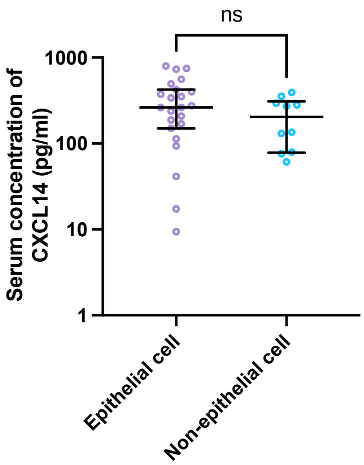

Figure S4. Scatter plots of CA125 and chemokine concentrations in different malignant cell types: epithelial cell type (n = 23) and nonepithelial cell type (n = 10). *p <0.05; not significant (ns). The lower and upper whiskers are the interquartile ranges, and the middle line is the me
